# Evaluations of Muscular Strength, Ability to Balance and Health Status in Prisoners during COVID-19

**DOI:** 10.3390/ijerph18084316

**Published:** 2021-04-19

**Authors:** Stefano Moffa, Angelica Perna, Alessandro Cattolico, Carmine Sellitto, Antonio Ascione, Domenico Tafuri, Germano Guerra, Angela Lucariello

**Affiliations:** 1 Department of Medicine and Health Sciences “Vincenzo Tiberio”, University of Molise, Via F. De Santis, 86100 Campobasso, Italy; stefano.moffa@unimol.it (S.M.); angelica.perna@unimol.it (A.P.); germano.guerra@unimol.it (G.G.); 2 Department of Mental and Physical Health and Preventive Medicine, Section of Human Anatomy, University of Campania “Luigi Vanvitelli”, 80138 Napoli, Italy; dottorcattolico@gmail.com (A.C.); carmine.sellitto@tiscali.it (C.S.); 3 Department of Educational Sciences, Psychology, Communication, University “Aldo Moro”, Piazza Umberto I, 70121 Bari, Italy; antonio.ascione@uniba.it; 4 Department of Sport Sciences and Wellness, University of Naples “Parthenope”, Via Medina 40, 80133 Naples, Italy; domenico.tafuri@uniparthenope.it

**Keywords:** prisoners, fitness levels, health status, COVID-19

## Abstract

Recent events in prisons during the COVID-19 pandemic showed how the health situation and overcrowding in prisons are a source of high risk to the health and physical and mental well-being of the prison population and how this has become an important medical problem. The original purpose of this study, which was initially planned to last 6 months, was to examine the effects of a training program on cardio-respiratory capacity, resistance to dynamic strength of the upper and lower body and muscle mass. Following the COVID-19 pandemic, the purpose was subsequently modified by highlighting whether and which deficiencies occurred as a result of the absence of physical activity. Forty adult men between 35 and 55 years of age with more than 1 year of detention were selected and randomly divided into two groups: the experimental group and control group. The fitness training protocol of the experimental group consisted of three weekly sessions lasting 90 min, while control group subjects followed a walk of 30–60 min three days a week without running or resistance training. The unpaired and paired t-tests revealed significant effects of both health status and fitness level (*p* < 0.05; *p* < 0.01) on group training. The results of this research show that prisoners can improve their fitness and health through participation in physical education programs. This conclusion is especially important for prisoners who have to serve very long prison sentences and who are at great risk of showing poor physical condition levels.

## 1. Introduction

Recent events in prisons in many Western countries during the COVID-19 pandemic showed how the health situation and overcrowding in prisons are a source of high risk to the health and physical and mental well-being of the prison population and how this has become an important medical problem [1,2,3,4,5].

Therefore, promoting healthy living habits among prisoners is very important for public health and for reducing health care resources and costs.

Many studies have shown that physical inactivity among prisoners has a higher incidence than the general population. This is relevant in view of the fact that physical inactivity is considered one of the most important risk factors for mortality worldwide. In addition, these individuals are at greater risk of developing chronic diseases (type 2 diabetes, hypertension, heart disease) that can be associated with muscle wasting and low functional capacity, where physical activity plays an important role as a protective factor [6].

In addition, recent research has shown that people living in prison in Western countries tend to develop mental illnesses and reduced psychological well-being, diseases in which physical activity has proven to have beneficial effects [7,8,9,10,11,12,13,14,15]. 

The realization of one’s physical and mental state and the ability to make good decisions for oneself is fundamental for the prisoner. All this improves the promotion of health that in prisons is very difficult [16,17].

It has been suggested that voluntary participation in sport can provide a means of raising awareness of healthy lifestyles and give an active form of learning that may be useful for this type of population [18]. 

Although prisoners are wary of a healthy life, the interest in participating in sport is often great [19,20]. Therefore, physical activity could play an important role in promoting health policy objectives. Furthermore, sport can provide great benefits for physical and psychological health in society [21].

Unfortunately, over time, little academic attention has been paid to the role of physical activity in the promotion of well-being among prisoners. However, studies have shown that people living in prisons are limited in the practice of physical activity due to the small size of prisons and the inability to perform physical exercises whenever they wish [22,23]. It would be desirable for criminal institutions to draw up special protocols allowing organized and guided physical activity through physical exercise.

Although previous research has shown the beneficial effects of exercise training on the health status and fitness level of prisoners, no study has focused on evaluating the duration time of these benefits. The COVID pandemic gave us the opportunity to verify this hypothesis. The original purpose of this study, which was initially planned to last 6 months, was to examine the effects of a training program on cardio-respiratory capacity, resistance to dynamic strength of the upper and lower body and muscle mass.

Following the COVID-19 pandemic, the purpose was subsequently modified by highlighting whether and which deficiencies occurred as a result of the absence of physical activity.

## 2. Methods

### 2.1. Subjects

All subjects participating in the study belonged to the Italian Maximum Security Prison of Larino (CB). Before beginning the study, informed consent was obtained from each participant and the study was approved by the Local Human Investigations Committee (protn. 0224-64-16/2019).

The study was conducted in accordance with the Declaration of Helsinki.

The subjects were selected among the 160 inmates of the Larino Prison (CB) with the following eligibility criteria: age between 35 and 55, male, detention for at least one year, and sedentary lifestyle. Eligible subjects were subjected to preliminary medical evaluation in order to identify medical conditions of such seriousness as not to allow participation in the proposed exercise program. Risk factors related to age, to particular symptoms, and to the use of drugs were evaluated. Finally, subjects with severe orthopedic, cardiovascular or respiratory conditions or those with a medical condition listed in the American College of Sports Medicine [24] as absolute contraindications to exercise were excluded. Forty of the total 46 inmates that had been originally selected met all the above-mentioned eligibility criteria and finally agreed to enter the study. All the selected subjects were randomly divided into two groups: the experimental group (no. 20; average age 35–55 years) and the control group (no. 20; average age 35–55 years).

### 2.2. Study Design

The subjects in the experimental group followed a fitness training of only 4 months (November 2019–February 2020) compared to the 6 months planned because the study stopped due to COVID-19 (from March 2020 to June 2020). 

**Figure 1:**
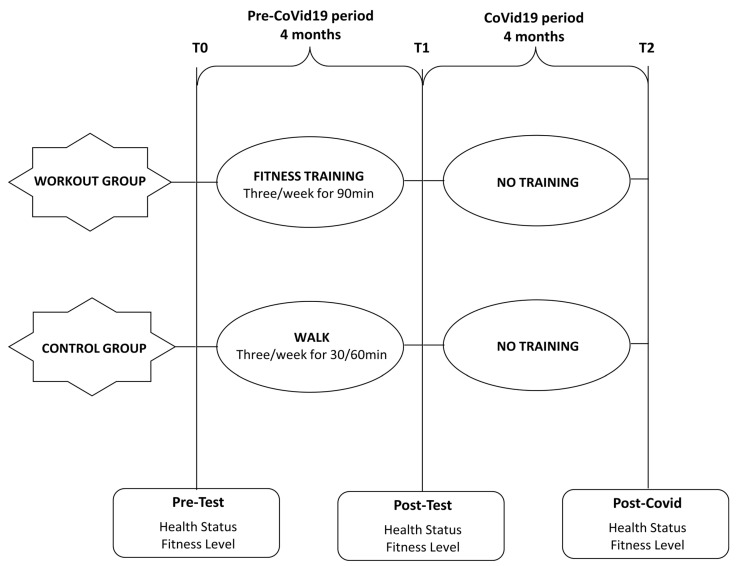
Study design.

All subjects in both groups were evaluated before and after the 4-month period and then post-COVID (July 2020) (Figure 1).

During the period of 4 months of training, the control group subjects followed a walk of 30–60 min three days a week without running or resistance training.

The physical training protocol of the experimental group consisted of 3 weekly sessions lasting 90 min each, as previously described [25]. All sessions were supervised by the same experienced investigator.

Each session started and ended with a warm-up and cooling-off period of 10 min. The central 70-min training session was divided into endurance and aerobic training. Endurance training included exercises involving the main muscle groups (chest press, leg curl, leg press, leg calf rise, abdominal crunch, low back extension, arm curl, arm extension, and lateral pull-down). 

All exercises were performed through the full range of motion normally associated with correct technique for each exercise. At the end of each series of endurance movements, stretching exercises of the muscles involved were performed. During the first month, subjects performed three series of exercises for large muscle groups (e.g., leg press) and small muscle groups (e.g., arm extension), with a resistance that allowed 12–15 repetitions (12–15 repetition maximum-RM) [25]. Subsequently, the resistance used was adjusted individually to allow the completion of 8–10 repetitions for three series of exercises for large muscle groups and two series of exercises for small muscle groups. The resistance used for each exercise was increased by 5–10% when the subject could perform the maximum repeats prescribed for series. Abdominal crunches and low back extensions were performed in two sets of 15–20 repetitions at the start of the program and in three sets of 20 repetitions at the end of it. At the beginning of the program, aerobic training consisted of pedaling on a bike ergometer for 20 min at 70% of the maximum heart rate expected by age (Fcmax = 220 − age). The duration and intensity of the sessions were gradually increased over the four-month period.

### 2.3. Testing Procedures

All subjects in both the experimental and control groups were tested for all health status variables for three days of the week in the morning and each measurement was repeated three times.

For all of them, standard anthropometric measurements, height and body weight were evaluated to calculate the body mass index (BMI), according to standard methods [26]. Resting blood pressure, systolic blood pressure (SBP) and diastolic blood pressure (DBP) and a pulse oximetry test (SpO2) were performed. SpO2 was used to measure the oxygen saturation of hemoglobin [27] and it was also used to detect significant metabolic diseases [28]. Subjects performed a complete spirometric test with Viasys Healthcare Flow Screen spirometer-Vyaire Medical, Lake Forrest, Illinois (USA).

Spirometric indices, cardiovascular fitness (CVF), forced expiration volume in 1 s (FEV1) and the Tiffenau index were used.

Only subjects with a Tiffenau index > 0.7 were included in the study. Patients with bronchial obstruction are considered below this value. 

### 2.4. Testing Procedures before and after the 4-Month Period and after the Post COVID Period

The following tests were given to assess how flexibility, muscle strength, endurance, balance, anaerobic strength, speed, and agility changed after the training period and after the COVID-19 period. 

The trial started in November 2019 (T0); from that moment to February 2020 (T1), both groups, control and experimental, followed the protocol, but in March, due to the pandemic, everything stopped. Therefore, we chose to evaluate the parameters again in July 2020 (T2), after a pause of four months.

The following tests were administered in subsequent order at the same time of the day and the measures were repeated three times.

The “2 min step test” is used to evaluate aerobic endurance. The subject stands up straight next to the wall while a mark is placed on the wall at the level corresponding to midway between the patella (kneecap) and iliac crest (top of the hip bone). The subject then marches in place for two minutes, lifting the knees to the height of the mark on the wall. 

The subject is permitted to slow down, stop or rest during the test, if he gets out of breath or feels tired, and then the subject should resume stepping as soon as he is able. The subject is informed when there is one minute remaining and when there are 15 s left. Subject should not talk while executing the test unless he has chest pain or dizziness. When the 2 min are up, the subject stops immediately. The scoring records the total number of times the right knee reaches the tape level in two minutes [29]. 

We also registered the heart rate of the inmates 30 s after the end of the two minute step test, counting the number of heartbeats in the following 30 s (we counted the beats of the heart from the 30th second after finishing the test to the 60th (HR30-60) second after the test) (Polar Ignite, Smartwatch with Advanced Heart Rate Detection-Polar Electro, Kempele, Finland). This gave us the possibility to evaluate the cardiovascular efficiency of the subjects and its variations. In the evaluation of the heartrate, according to the parameters previously reported [30], a lower HR after 30 s from the test reflects a better cardiovascular status.

Flexibility was evaluated with the “Sit and reach” test; in particular, this test measures flexibility of the lower back and hamstring muscles. This test was carried out using a step or a box with a ruler. The subject, after warming up, removed his shoes, and sat on the floor or a flat surface, with the legs extended in front of the body facing the box, with the toes pointing up; the ruler was positioned on the box, with the 0 lined up with the edge of the box. The subject, with hands on top of each other (tips of the middle fingers even), and palms down, had to reach forward slowly, sliding the hands on the ruler as far as possible. We measured the distance from the 0 in centimeters; if hands stopped before the 0, we had a negative measure; if the subject, with his hands, overstepped the 0, we had a positive measure [31,32].

The “One min half sit-up test” was considered as an indicator of abdominal muscular strength; for this test, the subject laid supine with the knee joint at right angles (90°) and the hands held across the chest; the subject performed as many half sit-ups as possible in 1 min [33].

The “Push-Up test” is an indicator of strength and muscle resistance from the top of the body and in particular of shoulders. For this test, each subject performed as many push-ups as possible in 1 min [34].

The “Arm Curl test” measures upper body strength and endurance. The aim of this test was to do as many arm curls as possible in 30 s. This test was conducted on the dominant arm side (or stronger side). Inmates used 8 lb dumbbells [35,36].

The “Stork balance test” requires the person to stand on one leg for as long as possible. The purpose is to assess whole body balance ability. The subject without shoes on placed their hands on their hips, then placed their non-supporting foot against the inside knee of the supporting leg. The subject raised their heel to balance on the ball of the foot. The stopwatch was started as the heel was raised from the floor and stopped when the raised foot touched the ground [37]. 

The “10 × 5 m Shuttle Test” is a measure of speed and agility. Participants ran back and forth for over 5 m, for a total of 50 m. To perform the Shuttle 10 × 5 test, we used two parallel lines placed five meters from each other. Subjects started with one foot on the line. When instructed by the timer, the subject ran to the opposite line, turned around and returned to the starting line. This is repeated five times without stopping (covering 50 m in total). At each marker, both feet must completely cross the line [38].

### 2.5. Statistical Analyses

The unpaired t-test and paired t- test were performed using Sigma Plot Software (Systat Software Inc., San Jose, CA, USA). The first one was used to compare the two groups at the base line. The paired t-test was used to analyze changes in inmate performance and comparing baseline results with results obtained after 4 months of training. In addition, these results were compared with those obtained after the stop caused by the COVID-19 pandemic. The changes were considered statistically significant for *p* < 0.05.

## 3. Results

No major side effects or major health problems were observed in the subjects in both groups during exercise. Subjects in the training group were satisfied with the results of the study and reported that they intended to continue the training program alone after the COVID-19 stop period. 

The unpaired t-test did not show statistically significant differences between the two groups at baseline.

Statistically significant changes were found for the group that practiced the training program, while for the control group, there were no statistically significant changes in the test results, but statistically significant improvements were noted in some parameters of the health status. Considering each dependent variable of health status, the paired t-test showed a significant effect from T0 to T1 for both groups. In particular, for the workout group, BMI (*p* < 0.05), SBP (*p* < 0.01), DBP (*p* < 0.01), SpO_2_ (*p* < 0.05), CVF (*p* < 0.01), and FEV1 (*p* < 0.01) showed this improvement. Meanwhile, for the control group, only SPB (*p* < 0.01), DBP (*p* < 0.05), FEV1 (*p* < 0.01) and TI (*p* < 0.01) showed this improvement.

Additionally, for the T1-T2 period, the paired t-test showed a significant effect for both groups. For the workout group, BMI (*p* < 0.05), SBP (*p* < 0.01), DBP (*p* < 0.01), SpO_2_ (*p* < 0.05), CVF (*p* < 0.01) showed a statistically significant change approaching the initial values recorded at T0. Meanwhile, for the control group, BMI (*p* < 0.01), CVF (*p* < 0.01), FEV1 (*p* < 0.05) showed statistically significant worsening changes compared to T1 and, in two parameters (CVF and FEV1), even below the values at T0 (Table 1).

Considering fitness level, the paired t-test showed significant changes from T0 to T1 only for the workout group for the 2 min step test (*p* < 0.01), heart rate recovery (*p* < 0.01), sit and reach test (*p* < 0.01), one min half sit up test (*p <* 0.01), push-up test (*p* < 0.01), arm curl test (*p* < 0.01), stork balance test (*p* < 0.01), and 10 × 5 m shuttle test (*p* < 0.01).

The same thing did not happen for the control group; performances did not change in a statistically significant way from T0 to T1 for the 2 min step test (*p* = 0.07), heart rate recovery rate (*p* = 0.20), sit and reach test (*p* = 0.75), one min half sit up test (*p* = 0.86), push-up test (*p* = 0.09), arm curl test (*p* = 0.36), stork balance test (*p* = 0.26), and 10 × 5 m shuttle test (*p* = 0.14).

For the workout group, moreover, performances changed again in a statistically significant way after the 4-month stop period due to COVID-19 pandemic (from T1 to T2) for each test—the 2 min step test (*p* < 0.01) heart rate recovery (*p* < 0.01), sit and reach test (*p* < 0.01), one min half sit up test (*p* < 0.01), push-up test (*p* < 0.01), arm curl test (*p* < 0.01), stork balance test (*p* < 0.01), and the 10 × 5 m shuttle test (*p* < 0.01).

Even after the forced stopping due to the pandemic period (from T1 to T2), performances of the inmates belonging to the control group did not change in a statistically significant way for the 2 min step test (*p* = 0.051), heart rate recovery (*p* = 0.13), sit and reach test (*p* = 0.48), one min half sit up test (*p* = 0.60), push-up test (*p* = 0.74), arm curl test (*p* = 0.09), stork balance test (*p* = 0.11), and 10 × 5 m shuttle test (*p* = 0.37) (Table 2).

## 4. Discussion 

The COVID-19 pandemic has highlighted the problems related to the health situation and overcrowding in our prisons, emphasizing the unacceptable health and social conditions in which the inmates live. In order to combat COVID-19, further restrictions have been introduced in prisons, which have had a very strong impact on the rights of people already deprived of their freedom. People in prison eventually become part of society again, and this means that their health is a matter of public health. Therefore, recently in both Italy and Europe there has been a need for effective interventions in prisons to guarantee public health. The prison should therefore become a place of education regarding healthier living habits through targeted interventions in the prison population.

Numerous studies have shown that people in prison can improve their physical condition by participating in physical education training programs [14,19,21,24]. 

Our study, although the training took place only for 4 months compared to the 6 planned, showed statistically significant changes in the health status parameters of the group workout considered. From November to February, in fact, all the parameters examined, excluding the Tiffenau index, improved in a statistically significant way.

The fact that only the Tiffenau index did not change in a statistically significant way is explained by the fact that it is the ratio between CVF and FEV1: these two parameters both improved, and so their ratio remained almost constant. After the training period of the experimental group, we also found an important improvement in the value of SpO_2_ [39]. As already reported by other authors, SpO_2_ values represent an effective tool for detecting arterial diseases of the lower limbs [38] and are useful for predicting mortality in interstitial lung disease related to systemic sclerosis (ILD) [39,40].

Even from February to July, there were statistically significant changes, regarding BMI, SBP, DBP, SpO_2_ and CVF (all worsened); the improvement in FEV1 was only minimally preserved. This means that the stop in physical activity caused by the pandemic caused the subjects to lose the improvements acquired during 4 months of continuous training.

These results were not observed for the control group. Some of the parameters taken into consideration improved statistically significantly from November to February. These parameters are: SBP, DBP, FEV1, and Tiffenau Index. This means that even adding just half an hour of walking three times a week to their prison life can bring benefits, although not comparable to what you get with the three training sessions practiced by the group workout.

For the control group, in fact, even when we recorded an improvement in the status of the pressure values, both systolic and diastolic, there was no statistically significant improvement in the BMI, which represents an important index of well-being/health; saturation dide not improve statistically significantly and, at the spirometric level, there was an improvement in FEV1, resulting in a decrease in the Tiffenau index.

After the stop caused by the pandemic, the improvements in pressure values were maintained, which means that the benefits of walking on blood pressure values were maintained even in the months following the stop, while the BMI, CVF and Tiffenau index worsened.

As for the performance recorded by physical tests, the first fact that is highlighted by looking at Table 2 is that in February (T1), there was a clear improvement in the group that performed the workout. Based on this, we can say with certainty that, after the first 4 months of training, in the subjects belonging to the workout group, there was an improvement in the aerobic endurance (2 min step test), cardiovascular efficiency (HR30-60), flexibility (Sit and reach test), abdominal muscular strength (One min half sit-up test), shoulder muscular strength (Push-up test), upper arm strength (Arm curl test), balance ability (Stork balance test), speed and agility (10 × 5 m Shuttle Test).

We observed a significant increase in the aerobic capacity of the training group after the 4-month intervention. Although we did not directly measure VO_2max_, our results are of clinical significance, because we used an approved test to estimate aerobic fitness in adults [41]. Previous research has also indicated that aerobic capacity indirectly assessed during gradual tests is an independent indicator of survival rates for both diseased and normal individuals [42]. 

We noticed, during the step test, a decrease in HR response to higher workloads, showing an improvement in aerobic condition. On the contrary, as previously described, an increased HR response to high workloads could represent a prognosis for coronary heart disease [43]. Agility, evaluated with the shuttle test, is improved by exercises for muscle strength, coordination and dynamic balance, as previously demonstrated in the literature [44]. In addition, a significant improvement in dynamic upper body and abdominal strength was observed. 

All these improvements were partially preserved after the 4-month stop, as recorded in the July values; however, on average, the scores were still better than those recorded in November, even if, in some cases, they were very close to the starting values. This suggests that a stop of more than 4 months would most likely have led to results similar to those recorded at T0.

Otherwise, for the control group, the small improvements recorded at T1 have not always been maintained; on the contrary, in some cases the values have deteriorated at T2 compared to T0; in particular for this group, the values recorded with the tests in July were always worse than those recorded in November except in two cases: for the stork test and the 10 × 5 Shuttle test. The controls after the 4-month stop, however, recorded better average values, even if only slightly, regarding those recorded for T0. The benefits of just walking carried out by the control group therefore concern balance, agility and speed and are kept even after long periods of no exercise, such as that caused by the COVID-19 pandemic. Finally, as noted, in order to achieve good aerobic capacity that is useful for maintaining optimal health and for maintaining ideal calorie consumption, an appropriate training program must be chosen [45].

Our study is not without its methodological limitations. Participation in the program was low because the detainees were heavily unmotivated due to their condition, and in addition, the subjects recruited were relatively young (aged 35–55). In addition, no direct evaluation of maximum aerobic capacity (VO_2max_) or muscle mass was possible. To measure these parameters, we would have to use equipment not present in the prison and, unfortunately, the inmates were not allowed to leave the building. 

A further limitation of the study was the lack of evaluation of biochemical and hematological parameters. In fact, several studies report significant changes in glucose, total cholesterol and triglycerides levels [25,45]. Finally, it would have been useful to verify if the physical activity proved to be an effective strategy for improving the general mental health of the inmates using a valid questionnaire.

However, the positive results obtained with regard to both health and physical parameters, even if with such a small number of subjects, reflect the great potential of exercise interventions to improve the health status of incarcerated people.

## 5. Conclusions

The results of this research show that prisoners can improve their fitness and health through participation in physical education programs. This conclusion is especially important for prisoners who have to serve very long prison sentences and who are at great risk of showing poor physical condition levels.

The quality of life of prisoners in prison is rather mediocre, and therefore the creation of specific training programs aimed at increasing this aspect could be accompanied by other strategies to improve the mental state of the subjects and have a greater impact.

In fact, one of the most common problems in prisons is the use of drugs that cause multiple mental disorders. Physical activity could therefore be used as a rehabilitative “therapy” for these subjects, improving mood and stress, and may be considered a distracting pastime.

In order to improve the behavior, mood and physical and mental well-being of prisoners, it is suggested that these activities are carried out, where possible, in groups.

Another important result of this study is the fact that none of the stakeholders left the program and that no side effects were found. Therefore, we suggest that programs of physical activity are supervised by an expert in order to avoid prisoners possibly incurring injury caused by the improper carrying out of a physical exercise.

Finally, the fact that the control group showed slight improvements in health parameters and in those concerning balance, speed and agility following a simple walk performed three times a week could suggest the utility of physical exercise outdoors, if possible, or indoors, even without the use of specific equipment.

## Figures and Tables

**Table 1:** Pre and post values for health status variables of workout group and control group shown as mean ± DS.

	WORKOUT GROUP			CONTROL GROUP		
	November 2019	February 2020	July 2020 (Post Covid-19 Stop)	November 2019	February 2020	July 2020 (Post Covid-19 Stop)
	Mean ± SD	Mean ± SD	Mean ± SD	Mean ± SD	Mean ± SD	Mean ± SD
BMI (kg/m²)	28.1 * ± 1.71	27.75 ^†^ ± 1.94	28.25 ± 1.68	27.9 ± 2.19	27.6 ^††^ ± 1.98	28.1 ± 2.04
SBP (mmHg)	123.75 ** ± 9.43	116.15 ^††^ ± 8.73	123.05 ± 8.03	123.6 ** ± 8.72	122.1 ± 8.49	122.9 ± 8.87
DBP (mmHg)	81.8 ** ± 6.97	76.65 ^††^ ± 6.42	81 ± 6.92	79.85 * ± 10.81	79 ± 10.17	79.6 ± 10.53
SpO_2_(%)	97.2 * ± 1.64	98.05 ^†^ ± 1.19	97.55 ± 1.46	97.1 ± 1.51	97.2 ± 1.5	97.3 ± 1.52
CVF (L)	4.59 ** ± 0.29	4.66 ^††^ ± 0.29	4.54 ± 0.3	4.73 ± 0.37	4.74 ^††^ ± 0.37	4.68 ± 0.36
FEV1 (L)	3.9 ** ± 0.28	3.97 ± 0.28	3.91 ± 0.27	4.04 ** ± 0.43	4.09 ^†^ ± 0.04	4.04 ± 0.41
TIFFENAU INDEX (%)	0.84 ± 0.02	0.85 ± 0.02	0.86 ± 0.02	0.85 ** ± 0.04	0.86 ± 0.04	0.86 ± 0.04

** *p* < 0.01 compared to February 2020; * *p* < 0.05 compared to February 2020; ^††^ *p* < 0.01 compared to July 2020; ^†^ *p* < 0.05 compared to July 2020 (BMI: body mass index; SBP: systolic blood pressure; DBP: diastolic blood pressure; SpO_2_: pulse oximetry test; CVF: cardiovascular fitness; FEV1: forced expiratory volume in 1 s).

**Table 2:** Pre and post values for fitness level variables of workout group and control group shown as mean ± DS.

	WORKOUT GROUP			CONTROL GROUP		
	November 2019	February 2020	July 2020 (Post Covid-19 Stop)	November 2019	February 2020	July 2020 (Post Covid-19 Stop)
	Mean ± SD	Mean ± SD	Mean ± SD	Mean ± SD	Mean ± SD	Mean ± SD
2 Minutes Step Test (n)	38.85 ** ± 6.01	42.45 ^††^ ± 5.96	39.6 ± 5.66	39.35 ± 5.72	40.15 ± 5.57	39.2 ± 5.48
HR 30-60	45.25 ** ± 4.1	41.95 ^††^ ± 3.97	45.1 ± 4.26	44.35 ± 4.27	45.05 ± 4.71	4.55 ± 5.48
Sit and reach (cm)	0.8 ** ± 4.17	2.85 ^††^ ± 3.82	1.7 ± 3.86	0.65 ± 4.95	0.71 ± 4.8	0.63 ± 4.63
Half sit up test (n)	24.3 ** ± 5.54	28.9 ^††^± 5.26	24.6 ± 6.01	25.25 ± 6.52	25.4 ± 6.32	24.7 ± 6.13
Push up test (n)	28.45 ** ± 6.15	35.55^††^± 5.35	29.7 ± 5.43	29.55 ± 8.11	29.15 ± 7.85	29.1 ± 8.01
Armcurl test (n)	26.85 ** ± 4.39	31.9 ^††^ ± 4.5	28 ± 4.1	24.9 ± 5.09	25.15 ± 5.26	24.8 ± 4.67
Stork test (s)	30.3 ** ± 6.36	34.35 ^††^ ± 6.26	30.45 ± 6.24	30.3 ± 6.36	31.1 ± 5.97	30.8 ± 6.14
10 × 5 shuttle test (s)	25.5 ** ± 3.84	22.8 ^††^± 2.89	7.25 ± 3.38	25.5 ± 3.84	25.2 ± 3.67	25.3 ± 3.84

** *p* < 0.01 compared to February 2020; ^††^ *p* < 0.01 compared to July 2020.
